# Quality of life in children and adolescents with migraine: an Austrian monocentric, cross-sectional questionnaire study

**DOI:** 10.1186/s12887-019-1537-0

**Published:** 2019-05-24

**Authors:** Lydia Stella Koller, Susanne C. Diesner, Peter Voitl

**Affiliations:** 1 First Vienna Pediatric Medical Center, Donau-City Straße 1, 1220 Vienna, Austria; 2 Sigmund Freud University Vienna, Freudplatz 1, 1020 Vienna, Austria

**Keywords:** Migraine, Quality of life, Children, Adolescents, Physical, Emotional, Social, Economical, School

## Abstract

**Background:**

Migraine is a disabling primary headache disorder that occurs in about 10 % of children and might lead to a lower quality of life. There are several possible migraine triggers in a patient’s environment, which should be avoided where possible.

The objective of this Austrian monocentric study was to identify migraine triggers and the areas, in which children and adolescents with migraine have a lower quality of life than healthy, headache-free children.

**Methods:**

In this cross-sectional, questionnaire study, 76 children from ages 8 to 17 years were included. Thirty-seven were classified as migraineurs, 39 as non-migraineurs. Participants filled in a questionnaire surveying the areas of physical, socio-economic and school functioning. Migraineurs further answered migraine-specific questions.

**Results:**

The study included 33 (43.4%) males and 43 (56.6%) females. Median age was 13.00 (10.00–16.00) years. Average age of onset for migraine was 9.22 ± 3.34 years. Non-migraineurs skipped trendwise fewer meals (p.adjust = 0.108) and exercised more often (p.adjust = 0.108). In socio-economic functioning, the father’s nationality being Austrian might be related to migraine (p.adjust = 0.108). Children with migraine had a significantly lower quality of life in school functioning (PedsQL 4.0 questionnaire, p.adjust = 0.04) and had significantly less often “good” grades than children without migraine (p.adjust = 0.048).

**Conclusion:**

Children with migraine show a reduced quality of life in the areas of physical, socio-economic and school functioning.

## Background

Migraine is categorised as primary headache disorder [1, 2], which is divided into “migraine without aura”, a clinical syndrome characterised by a pulsating headache and associated symptoms of nausea, vomiting, photophobia, phonophobia as well as motion sensitivity and “migraine with aura”, consisting of transient focal neurological symptoms, mostly visual or sensory, which precede or accompany the headache [1, 2]. Migraine can develop at all ages [2], and affects 7.7 to 9.1% of children and adolescents [3, 4].

Population based studies have identified a number of physical and emotional triggering factors, which can induce a migraine attack. Physical factors that can trigger migraine are age [5–7], comorbidities (i.e. atopic disorders [8], food intolerances and allergy [9], obesity [10, 11], sleep disorders (sleep walking, sleep talking, nightmares, bruxism) [12, 13]), caffeine consumption [9, 14], skipping meals [15], alcohol consumption [9], immobility [10], weather [16, 17], noise, menstruation [16] and smoking [10]. The effect of daily fluid intake [14] and use of digital media is questionable [18–20]. Emotional factors include emotional stress from family [21] or general unhappiness [6], but also psychiatric disorders, such as depression and anxiety, as reviewed in an article by Bicakci [22]. Social factors that influence migraineurs are having fewer friendships compared to their siblings [23], furthermore the risk of migraine is increased by lack of empathy during headache attacks from parents, family quarrel [21] as well as high expectations from parents [6]. Further factors are sexual or emotional abuse (bullying) [24, 25], unfair treatment by teachers [26] and low economic status [27]. School stress is the biggest predicator for migraine [28]. In children with migraine absenteeism is increased, whilst school performance is reduced [28]. Severity, duration and frequency of migraine attacks can cause poorer performance in school [29].

With regard to these data it becomes obvious that migraine affects already pediatric patients in various areas of their lives. We aimed, therefore, to analyse the impact of migraine on quality of life in young children and adolescents in an Austrian single-center based study.

## Objectives

The main objectives of this study were:

1. To substantiate that, in a single-center cohort, Austrian children and adolescents with migraine have a lower quality of life than healthy controls in specific areas of life.
2. To collect data concerning features of migraine attacks and treatment in this Austrian cohort
3. To identify lifestyle factors that might influence the prevalence of migraine and to reveal how migraine affects the patient’s life, in the areas of: (a) physical functioning, (b) economic status and social functioning, (c) school functioning.

## Methods

### Study design and population

This study was designed as a monocentric, cross-sectional questionnaire study. Ethical approval was applied for and granted by the local ethics committee of the Medical University of Vienna (1210/2016). Participants for this study were recruited in a Viennese pediatric outpatient clinic, the “First Vienna Pediatric Medical Center,” and were asked to fill in the questionnaire after giving written consent.

Children and adolescents diagnosed with migraine, 8 to 17 years of age, being under regular medical treatment at the pediatric outpatient clinic were asked to participate in this study. Age- and sex-matched pediatric patients, who do not suffer from migraine were enrolled in this study as control group. These healthy controls were pediatric patients, also recruited at the First Vienna pediatric medical center, who were at the practice due to health control check-ups, vaccinations or due to acute infections but without headache. Participants included as “migraineurs” had to fulfil the ICHD-3 beta criteria [1] of migraine. Exclusion criteria were other primary headaches or secondary headaches. Two types of questionnaires were handed out to the participants and asked to be filled in: (1) Questionnaire for children from 8 to 12 years of age, who could accept their parents support in filling in the questionnaire. (2) Questionnaire for adolescents from 13 to 17 years of age, which has some additional, age-typical questions and could be completed independently. The reason for a separate questionnaire for teenagers was on the one hand chosen due to questions regarding puberty and on the other hand due to the validated PedsQL™ questionnaire, which was also designed for children either younger or older than 13 years. At least 90% of the questionnaire had to be filled, for the questionnaire to be included into the evaluation. Data from other sources, such as health records or other medical results were not used for this study. Furthermore, written consent from participants of all ages and their parents was required.

### Questionnaire

The questionnaire is essentially designed by the author of the study. One part was used directly from the “Pediatric Quality of Life Inventory” (PedsQL™) questionnaire, version 4.0 [30], which has been validated and demonstrated good reliability in healthy and ill children and infants [31, 32]. The permission for use of the PedQL™4.0 questionnaire was obtained from Mapi Resarch Trust in February 2016.

The designed part is a survey of: (1) migraine features, (2) migraine triggering factors, based on results of earlier studies, (3) the influence of migraine on the patient’s life.

The part of “school functioning” contains questions directly from the “PedsQL 4.0” questionnaire [30]. The German-Austrian version 07/01 of the PedsQL™, version 4.0 child-report for children (8–12 years) [33] and teens (13–18 years) [34] was used, taking only the questions for “school functioning”, which are 5 items in total. All other dimensions of the PedsQL™, which includes 23 items, covering in addition to school functioning, physical (8 items), emotional (5 items) and social (5 items) dimensions were not used for this survey. The items of school functioning are scaled on a 5- point Likert Scale from 0 (never) to 4 (almost always). Items are then reversely scored and linearly transformed to a scale from 0 to 100 (with 0 = 100 and 4 = 0). The mean score is calculated from the sum of items over the number of items answered [35]. The higher the scores, the better the health-related quality of life.

In detail, the questionnaire collects the following parameters:

1. **General**
  - Age
  - Gender
  - How many siblings are there? Are they younger, older or both?
2. **Headache parameters**
  - At what age did the first migraine attack occur?
  - What type of medication and how often is it used in case of acute attacks and for prevention?
  - How often is the hospital visited per month due to an attack?
  - How often is the GP visited per month due to an attack?
3. **Physical functioning**

- Are there any problems or restrictions for all participants concerning diet in migraineurs, regarding physical exercise and migraine triggering situations?
- What are the participants’ habits when it comes to meal skipping, daily fluid intake and weekly physical exercising?
- How many hours per day are spent with digital media?
- Are there any problems with hobbies and migraine?
- Are there any migraine-accompanying symptoms, such as nausea, vomiting, photophobia, phonophobia, aura symptoms (visual, sensory, etc.) or hemiplegia?
- Are there any co-morbidities, such as overweight, atopic disorders (asthma, rhinitis, dermatitis), allergies, food intolerance, epilepsy, sleeping disorders, as well as psychic comorbidities, such as depression or anxiety?
- Did or do any episodic syndromes still occur, such as abdominal pain, cyclic vomiting, benign paroxysmal vertigo or benign paroxysmal torticollis?

4. **Emotional functioning**

- Does the patient have a lower subjective self-esteem?
- Is the patient in psychological care?

5. **Socio-economic functioning**

- What are the parents’ citizenships?
- What are the parents’ educations?

1. compulsory school, A-levels, apprenticeship/college, university

c. Where does the patient reside (Vienna or another municipality)?
d. What size is the flat/house in square meters?
e. Is there any lack of understanding concerning migraine attacks, coming from:

2. Family, including parents and siblings
3. Friends
4. School colleagues
5. Teachers

f. When having an attack is aid provided by the individuals listed in 5.e. above?
g. Who gives appreciation? (see 5.e.)
h. Who applies pressure? (see 5.e.)

6. **School functioning.** This part is copy right protected by Mapi Research trust and therefore can directly be accessed online [30, 33, 34]. Additional self-designed questions of school functioning included:

- Are there more migraine attacks before or after school tests?
- Does the patient have good grades in school?
- Would the grades be better without the migraine?

### Questionnaire for adolescents of age 13–17 years

Basically, the same parameters are asked, with the following supplement:

- **Ad 3. “physical functioning”:**
  - How much coffee is consumed daily?
  - Does the patient smoke?
  - Are there any problems with migraine in leisure time?
    1. going-out: alcohol, drugs, clubbing, concerts, cinema
    2. partner: sex (problems with sex, effect of sex on migraine), effect of contraceptives on migraine
  - Are there any problems with migraine associated with menstruation?
- **Ad 5. “socio-economic functioning”:**
  - Evaluating the same questions as the questionnaire for 8–12 years (see socio-economic functioning), but also including “partners” as reference person.
- **Ad 6. “school functioning”:** [34]
  - Does migraine influence the choice of a specific education?

### Statistics

The raw data was compiled in Microsoft Excel and analysed using IBM SPSS Statistics (IBM Corp., NY, version 25.0). Missing data was excluded from the calculation. Graphics were done with GraphPad prism software.

Statistical evaluation was applied to the total number of study participants, as well as the subgroups of migraine, “migraineur” and “non-migraineur”. Father’s nationality and education each missed data from 3 participants, therefore the total number of subjects in these questions was only 73.

Descriptive statistics were performed and parameters described as categorial (absolute and percentage frequencies (n (%)), as well as continuous (mean and standard deviation (mean ± SD) or median and IQR (median (IQR)), when not normally distributed). Percentages represent the frequency in our patient cohort, but are, due the small sample size and the monocentric design of the study, not extrapolatable to the general Austrian population.

Significance was tested between the groups of migraineurs and non-migraineurs: To determine statistical significance for categorial variables, a χ^2^-test was performed or, in case of expected frequencies less than 5, Fisher’s Exact Test was used. Normal distribution was determined with the Shapiro-Wilk-Test. Almost all continuous variables were *not* normally distributed. Therefore, the non-parametric Mann-Whitney-U-Test was conducted to establish statistical significance. Correction for multiple testing (Table 1) was conducted using Bonferroni-Holm method. The alpha-level was set at 0.05, the two-sided *p*-value at <0.05.

**Table 1:** Significant results

*Variable*	*Total* *n* = 76	*Migraineur* *n* = 37	*Non-Migraineur* *n* = 39	*P-value*	*Adjusted p-value* ^*b*^
Meal skipping					–
None	32 (42.1%)	11 (29.7%)	21 (53.8%)	**0.039**	**0.108** ^a^
Physical exercise per week ≥3 times	34 (44.7%)	12 (32.4%)	22 (56.4%)	**0.036**	**0.108**
Nationality father Austria^c^	60 (82.2%)	33 (91.7%)	27 (73.0%)	**0.037**	**0.108**
PedsQL (mean score)	75.00 (60.00–83.75)	70.00 (55.00–80.00)	80.00 (60.00–85.00)	**0.008**	**0.04**
Subjectively good grades	57 (75.0%)	23 (62.2%)	34 (87.2%)	**0.012**	**0.048**

Data are described as n (%) for categorial variables and median (IQR) for continuous variables

Tests for significance are χ^2^-test/ Fisher’s Exact Test (categorial variables) or Mann-Whitney-U-Test (continuous variables); *p*-value <0.05 was considered as significant

^a^Fisher’s Exact Test

^b^*P*-value was adjusted for multiple testing using Bonferroni-Holm method

^c^*n* = 73, as 3 values were counted as missing data

A binomial logistic regression was performed to determine whether there is a relationship between migraine (dependent variable) and several independent variables. These variables were: age, age group, gender, meal skipping, parental (maternal, paternal) nationality, appreciation and pressure. Modelling assumes risk factors do not interact. A box-Tidwell test was executed to confirm a linear relationship between the dependent and independent variables. Variables were then tested for binomial logistic regression: First, “Omnibus-Test” (χ^2^-test) was examined for statistical significance. If significant, further evaluation of the data was performed. If not, analysis was terminated. Further evaluation involved analysing significance of the coefficient (Wald-Test) and odd’s ratio.

## Results

### Demographic parameters of study population

A total number of 76 pediatric participants was included in the study. The migraine group accounted for 37 (48.7%), the non-migraineur group 39 (51.3%). Age group 8 to 12 years consisted of 34 (44.7%) participants, of whom 14 (37.8%) were migraineurs, 42 participants (55.3%) in age group 13 to 17 years, of whom 23 (62%) were migraineurs (*p* = 0.239). The study contained 33 (43.4%) male and 43 (56.6%) female participants. Eighteen (48.6%) of migraineurs and 25 (64%) of non-migraineurs were female (*p* = 0.174). Median age at time of questionnaire was 13.00 years (10.00–16.00). Average age of onset in migraine was 9.22 ± 3.34 years.

### Treatment, medical care and triggering factors of migraineurs

**Fig. 1:**
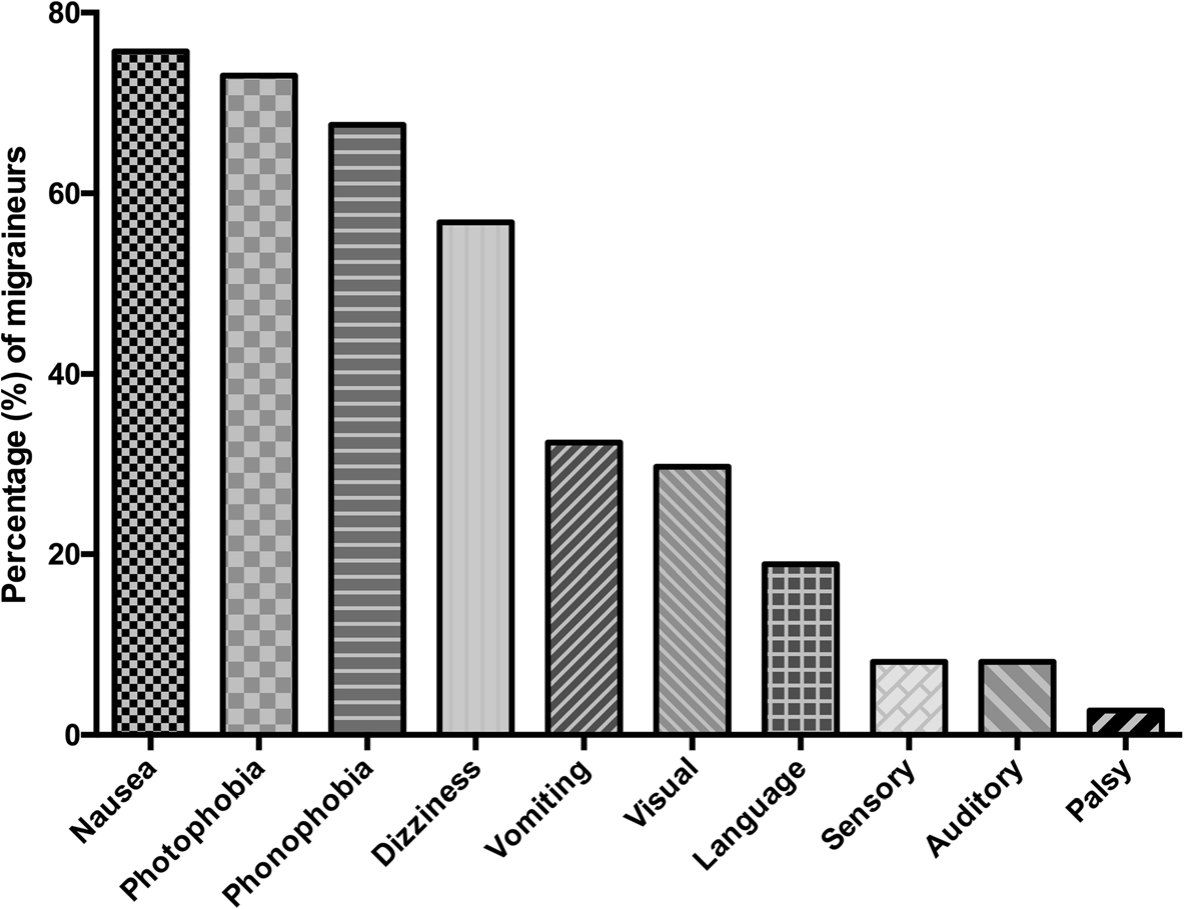
Migraine-accompanying symptoms. The percentages of symptoms accompanying migraine in migraineurs are depicted

**Fig. 2:**
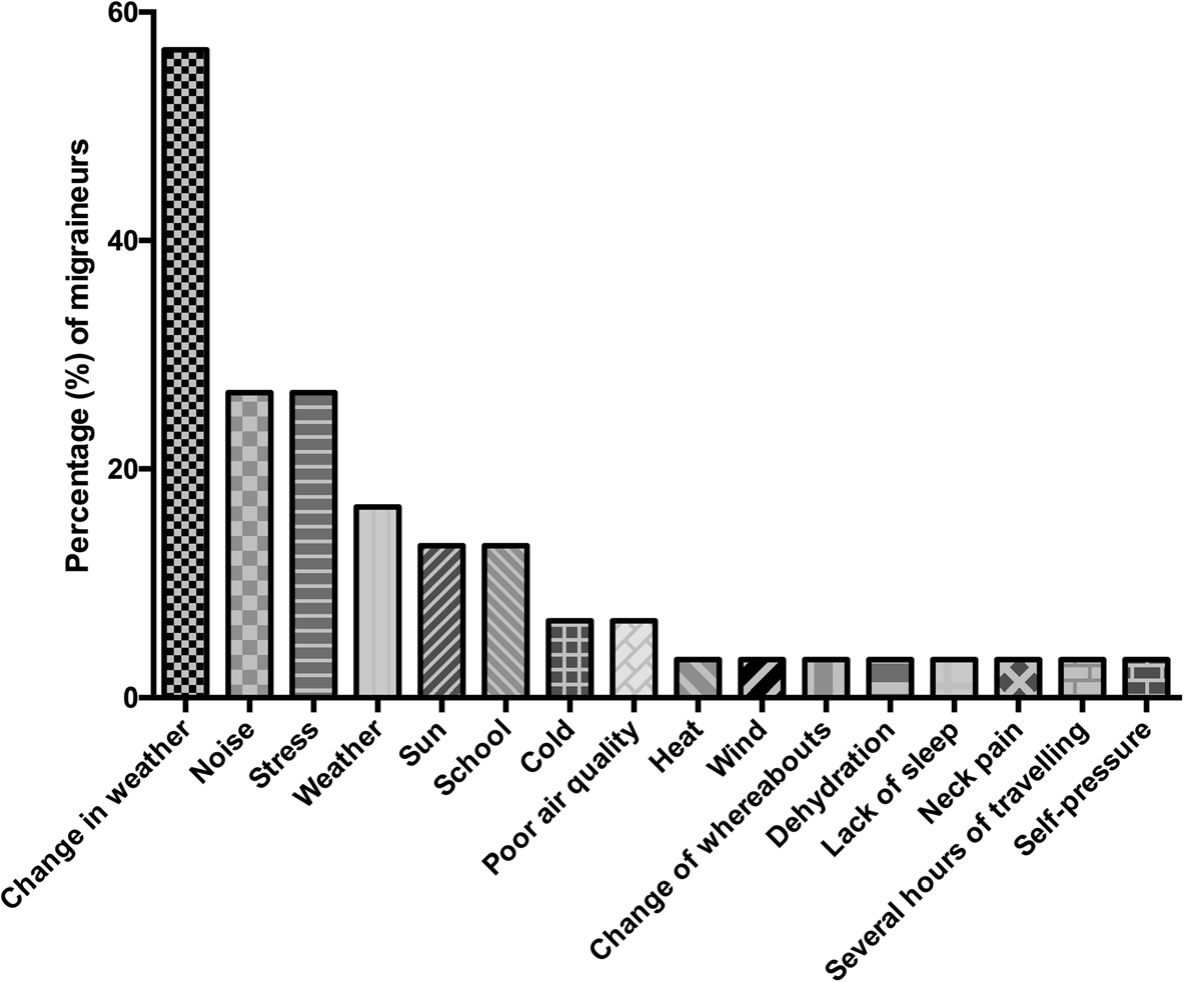
Migraine triggers. Certain situations were reported by migraineurs to trigger an attack. Percentages of migraineurs are given

**Fig. 3:**
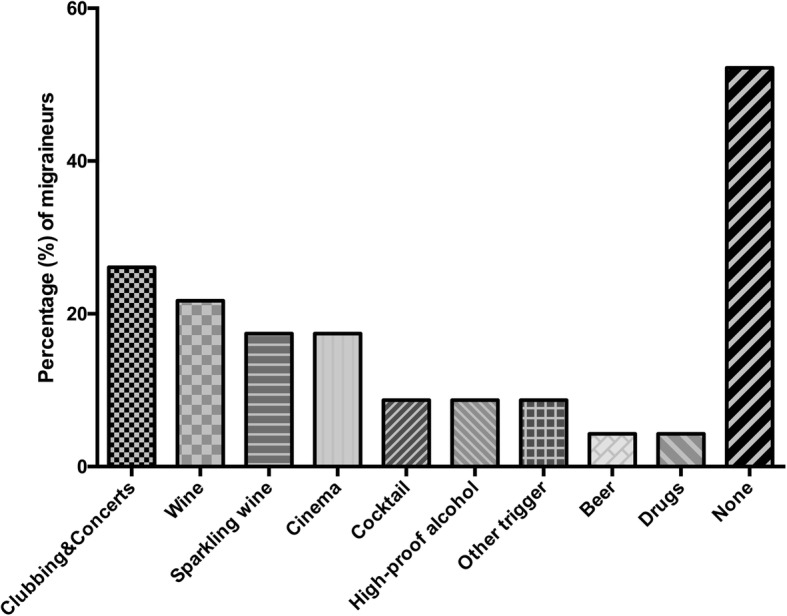
Migraine triggers in adolescents. Percentages of migraine triggers when going-out are shown in 13 to 17- year-olds

In the group of migraineurs (*n* = 37), migraine accompanying symptoms (Fig. 1) included nausea, photo- and phonophobia and dizziness. “Visual aura” was the most frequent aura symptom, in 11 participants (29.7%) and was depicted with blurred vision (*n* = 5, 45.5%), flickering (*n* = 3, 27.3%), spots (*n* = 3, 27.3%), light circle (*n* = 1, 9.1%) and limited field of view (*n* = 1, 9.1%). 33 (89.2%) patients used medication, 32 (86.5%) only in case of migraine attack while 2 (5.4% of) patients used preventative medication as well. Substances indicated as used “attack medication” were ibuprofen by 18 (56.3%), paracetamol by 8 (25.0%), mefenamic acid by 7 (21.9%), acetylic-salicylic acid by 5 (15.6%), caffeine by 2 (6.3%), and metamizole and naproxen each by one (3.1%). Substances used for “preventative medication” were propranolol (*n* = 1) and zonisamid (*n* = 1). None of the participants needed medical care at an emergency unit or outpatient clinic on a regular basis, but in total 5 (13.5%) patients have visited the hospital and 13 (35.1%) patients have visited the GP due to migraine attacks since they were diagnosed. Triggering situations were indicated in 30 (81.1%) of migraine responses (Fig. 2). Triggering hobbies were noted in 6 (16.2%) of migraineurs, mostly use of digital media. Triggers for going-out mainly included concerts and clubbings, as depicted in Fig. 3. In 13 to 17-year-olds (Fig. 3), problems with sex were stated by 1 (4.3%) of 23 migraineurs, sex influenced migraine for the better in three (13.0%). A bad influence of the contracepting pill was reported by one (4.3%) of 13 to 17-year-olds, four (17.4%) indicated a negative influence of menstruation.

### Physical functioning

Non-migraineurs skipped trendwise fewer meals than migraineurs (χ^2^ (1) = 4.530, *p* = 0.039, p.adjust = 0.108, for details see Table 1). Breakfast, morning snack, afternoon snack and dinner did not show any significance. Dietary restrictions, daily fluid intake, coffee per day or smoking did not differ between the groups.

Impairment in physical exercising due to migraine was stated by eleven (29.7%) migraineurs. A significant relationship was found between physical exercise per week and migraine (χ^2^ (3) = 8.091**,** *p* = 0.045): Migraineurs exercised less often ≥3 times per week than non-migraineurs did (χ^2^ (1) = 4.415, *p* = 0.036, p.adjust = 0.108).

Though migraineurs used digital media more often, it resulted in no significant difference, only mobile phone (U = 563, *p* = 0.097) showed a trend towards significance, with migraineurs spending more median time with their mobile phones.

### Socioeconomic functioning

There was no relationship between maternal nationality and migraine (χ^2^ (1) = 0.781, *p* = 0.377), but their might been between paternal nationality and migraine (χ^2^ (1) = 4.356, *p* = 0.037, p.adjust = 0.108): Migraineurs more often had Austrian fathers than non-migraineurs.

Maternal and paternal education did not have a relationship to migraine. Neither did residence with Vienna (57.9%), or federal state, Lower Austria (38.2%) and Tyrol (3.9%). Migraineurs did not occupy a significantly smaller living space (U = 719, *p* = 0.979).

There were no significant results in appreciation or pressure by parents, siblings, friends, school colleagues, or teachers. Migraineurs received appreciation most frequently from parents in 36 cases (97.3%) and if pressure was applied, it was mostly by teachers in 17 cases (45.9%).

### School functioning

More than a third (12 out of 37, 32%) of migraineurs think that their grades would be better without migraine. In 8 to 12-year olds, one of 14 (7.1%) suffered from headaches before a school test, two (14.3%) afterwards. In 13 to 17-year olds, one of 23 (4.3% of cases) stated that migraine influenced the choice of education. This is underlined by the results of the PedsQL 4.0 (school functioning) questionnaire [30] showing a significantly lower score in school functioning of migraineurs than non-migraineurs (U = 468, *p* = 0.008, p.adjust = 0.04). Migraineurs did report less good grades, represented by subjective evaluation of school achievements, than non-migraineurs (χ^2^ (1) = 6.338, *p* = 0.012, p.adjust = 0.048).

### Binomial logistic regression

To determine the likelihood of certain variables causing participants to have migraine, binomial logistic regression was conducted. None of the tested variables, except for paternal nationality, showed a significant result in binomial logistic regression and therefore a causal relationship towards migraine in our cohort.

“Paternal nationality” had significant results (χ^2^ (1) = 4.564, *p* = 0.033), the coefficient of “Austria” being significant (Wald test, *p* = 0.047). Austrian fathers were 4.07 times more likely in migraineurs than fathers from other countries. Furthermore, children with fathers from “other country” had 75.5% lower odds to have migraine than children with fathers from Austria.

None of the other variables showed significant results in the Omnibus-Test, therefore further analysis was not performed.

## Discussion

Migraine represents a common health problem in our society and can already start early in life, affecting children and adolescents. In literature, data on characteristics, triggering factors and impact of migraine on quality of life are based on epidemiological population studies. However, as far as we know this is the first report on pediatric migraine patients in an Austrian cohort. To identify the impact of the disease on the patients’ quality of life with regards to physical and socioeconomic functioning we conducted a monocentric, cross-sectional questionnaire study on children and adolescent migraine patients and compared the results to age and sex-matched healthy non-migraineurs. Due to the monocentric approach, only 76 pediatric patients were enrolled in this study, which might explain some of the non-significant results being in contrast to reports in literature, such as a possible association of migraine and gender or age [36, 37].

In our study, we observed the most incisive effects of migraine on school performance and physical activity. School per se is not only a migraine triggering factor, but school performance is negatively correlated with the intensity of migraine, resulting in a lower average grade in migraineurs [29]. Migraineurs had a significantly reduced quality of life in the PedsQL™ 4.0 questionnaire and significantly less often stated having “good” grades at school compared to non-migraineurs. Further, more than a third of migraine patients thought that their grades would be better without migraine, about 20% having headache attacks occurring concomitant with school tests. In line with our results, several other studies found a significantly lower quality of life in migraineurs compared to non-migraineurs (PedsQL™ 4.0) [38–40]. It may be hypothesized that migraine mostly affects the area of school functioning, as especially severe migraine attacks may disable patients, sometimes for days, which renders accomplishing school tasks impossible.

With regard to physical functioning, our migraine patients exercised less often 3 or more times per week than non-migraineurs, which was accompanied by an impaired ability for physical exercising due to migraine in one third of migraineurs and which might be explained by the assumption that exercise might trigger a migraine attack [18, 41]. Spending less time with sports seemed to be associated with migraine in some studies [14, 42], even though others could not find any significant relation in physical inactivity and migraine [43]. Interestingly, the spare time migraineurs had due to reduced exercising time was not spent with digital media, although migraineurs tended to spend more time with their mobile phones than non-migraineurs. Bektas et al. found that migraineurs invested less time in TV watching and computer playing than non-migraineurs [42]. Whether or not this might be due to digital media as migraine trigger remains questionable and implies the need of additional studies on this topic in future.

Physical health of migraineurs is also affected with regards to dietary habits. A study by Bektas et al. confirms our results that migraineurs skip meals more often [42]. Other groups also reported on fasting and hunger as migraine triggers [18, 44]. This, however, was discussed controversially by other authors, who could not detect a correlation of migraine with students skipping meals [14]. A link between fasting, i.e. skipping meals, and migraines may be suspected, as paediatric migraineurs have reduced appetite compared to non-migraineurs, possibly provoked by physical (nausea in attack) or emotional reasons. Other factors promoting migraine attacks in our patients included most commonly weather conditions, noise and stress, but also cinema, clubbing and concerts. Stress and lack of sleep seemed to be the most common trigger factors in other studies, followed by noise and excitation, intense visual stimuli, such as visual fixation, lights and video games and weather [18, 39, 42–44]. It may be hypothesized that migraine is triggered by intense changes of physical factors in the environment, as well as the effect of stress on the patient’s body. Adolescent migraineurs might consider reducing or refraining from alcohol consumption when going out as alcoholic beverages trigger migraine attacks [43, 45], even if the relation between migraine and alcohol is controversially discussed in literature [14].

Finally, migraine influenced socio-economic areas in our patients. Interestingly, a relationship between father’s nationality and migraine was observed, as children with migraine had Austrian fathers significantly more often, which however was not found for maternal nationality. While Singh et al. described migraine prevalence to be significantly higher in children with native-born, non-Hispanic white parents than those with Hispanic, immigrational background [46], Bugdayci et al. reported on a higher headache prevalence in immigration families [37]. Therefore, assuming a relation between paternal nationality and migraine is questionable and needs to be addressed in future studies in more detail. Not only the parental nationality background but also educational and socio-economic status of the family might influence migraine in children. Parents with a low educational level have more often children with headaches [37], while family’s living space is not correlated with a higher headache prevalence. Our study did not obtain a significant result on paternal education or residence (Vienna or other federal state) and size of living space. Families are the major source of aid in an attack for migraineurs, who do not lack appreciation or are pressurized from the social environment. That this is not the case for other populations is underlined by contradictory results in literature. Some migraineurs felt a subjective lack of support by their siblings [47] and had significantly reduced scores in social relationship compared to healthy children, having a lower ability to communicate in school and social environment [48]. Whether there is an association between social relationships and migraine is hard to assess, due to conflicting outcomes from literature.

The main limitation of this study was the relatively small sample size, which however was based on the single center design of this study and which might be the explanation for some of the non-significant results. A larger number might have produced more significant results. In addition, the questionnaire itself may have addressed too many variables, as it tried to cover a lot of areas simultaneously. The phrase “headache” as a synonym for “migraine” was problematic, as a lot of healthy children did not recognise questions including this phrase as “migraine-specific” questions. Additionally, as young children and adolescents might not have understood all of the questions correctly and as their parents might reflect the children’s well-being and quality of life differently than the child experiences this might lead to a reporting bias. To minimize this bias, the authors assisted the families in filling in the questionnaire in those cases, where ambiguity existed. The recommendations to future researchers would be: (1) to focus on only one area of functioning to carve out key aspects of the variables, (2) to clarify misleading phrases and descriptions, (3) to enlarge the sample size.

## Conclusion

In this Austrian cross-sectional questionnaire study, pediatric migraineurs appear to have a reduced quality of life in physical, socio-economic and school functioning, compared to healthy children. Most prevalent migraine triggers were weather, stress, noise, alcohol, cinema, clubbing and concerts. In general, migraine triggers and implications of migraine on areas of life are controversially reported on in population-based studies. The non-significant results of our data might be partially explained by the small sample size, which is based on the monocentric approach of this study. However, our results implicate that pediatric migraine patients do not only need special medical attendance but support in other areas of life.

## Abbreviations

ADHD: Attention deficit hyperactivity disorder
ICHD: International classification of headache disorders
IQR: Interquartile range
OCD: Obsessive-compulsive disorder
PedsQL™: Pediatric Quality of Life Inventory
SD: Standard deviation
tTGA- antibodies: Tissue transglutaminase antibodies
